# Developing Korean children’s physical activity health literacy: Literate, Empowered, Active, Doer program (LEAD)

**DOI:** 10.1080/17482631.2019.1648940

**Published:** 2019-08-09

**Authors:** Seunghyun Baek, Okseon Lee

**Affiliations:** a Banghwa Elementary School, Seoul, South Korea; b Department of Physical Education, Seoul National University, Seoul, South Korea

**Keywords:** Health literacy, physical activity health literacy, elementary school, health program, physical fitness

## Abstract

Purpose: The purpose of this study was to describe children’s experiences of the impact of LEAD (Literate, Empowered, Active, Doer) health program on their physical activity health literacy and to describe the characteristics of the LEAD health program that influenced the children’s health literacy enhancement. Methods: Six fifth grade elementary school students (three boys, three girls) were selected as participants. The data were collected from open-ended questionnaires, in-depth interviews, observation, and student journal entries. The data analysis followed the procedure of qualitative content analysis, which consisted of transcription, encoding, and creation of themes. Findings: Findings revealed that the children’s physical activity health literacy was enhanced in four separate manners: (a) became smarter finders of health information, (b) became active agents in sharing and applying health information, (c) valued and expanded physical activity beyond PE class, and (d) understood health as a holistic concept. Regarding program characteristics that influenced students’ changes, three themes emerged: (a) providing specified contents in various physical activities to students (b) granting autonomy to students, and (c) building supportive environments for students.

## Introduction

School is generally regarded as an optimal setting to nurture students’ knowledge, skills, and attitude on the subject of health (Yu, Yang, Wang, & Zhang, 2012). This evidence indicates that the locus of responsibility in regards to children’s health statuses should be imposed significantly by the schools. In South Korea, the ultimate goal of physical education is to promote children’s ‘healthy and active lifestyles’ and to cultivate children’s character (Korean Ministry of Education, 2015). Moreover, ‘health’ is presented as one of the five key areas in the Korean National Curriculum of Physical Education (KNCPE) for developing students’ health management capacity and promoting healthy lifestyle, beyond the classroom (Korean Ministry of Education, 2015).

However, according to the national report, Korean adolescents’ participation in physical activity gradually declines after their childhood ends (Korean Ministry of Education, 2018), and their level of physical fitness also lowers (Lee, 2012). It implies that a growing number of students are becoming estranged from the physically active lifestyle, which leads to future engagement in unhealthy behaviours. Given that the rates of adolescents smoking, drinking, and drug abuse are increasing (Lee, 2010), the physical education in South Korea does not provide an adequate educational foundation to promote long-term, healthy lifestyles in its students.

In addition to the growing health risks suffered by these adolescents, teaching health in elementary PE class is a challenging task for Korean teachers. First, there has been a lack of resources in regards to proven content and methodology to support effective health education in elementary school. Second, since academic research on health and fitness are mainly focused on the physiological and medical aspects of health, teachers experience a considerable gap between the scientific knowledge and the application of it in their teaching contexts. Consequently, these practical difficulties suggest that elementary health education should be tailored solely to elementary school students’ developmental needs (Rhee & Jo, 2015).

It is evident that a conceptual approach to defining ‘health’ should be a focal point of deliberation ahead of any other issues, as there are many cases in which the term ‘health’ is used in a distinctive sense, both educationally and socially (Park, 2017). For example, those taking a biomedical approach (Ereaut & Whiting, 2008) define health as physical soundness without disease, while those taking an individual responsibility discourse (Griggs, 2015) focus on an individual’s efforts to manage a healthy lifestyle; these examples conflict with other approaches that emphasize the social and environmental factors influencing health. Along with a goal of developing well-rounded people, elementary physical and health education should be implemented with a holistic approach to health, embracing physical, mental, and social health as one (Griggs, 2015; World Health Organization, 1948). Furthermore, health domain in elementary PE should be educated in such a way that students can fully understand the meaning and importance of the holistic health concept and possess competence, knowledge, positive attitudes, and functions for healthy lifestyles; in other words, the focus cannot rest entirely on the outcome of fitness testing. In this regard, promoting ‘health literacy’ should be an important goal of health education in South Korea.

Health literacy has been established as a crucial aspect of health worldwide (Kickbusch, Pelikan, Apfel, & Tsouros, 2013). The concept of ‘health literacy’ was first used as a guideline for health education in the USA, and it was defined as ‘the cognitive and social skills which determine the motivation and ability of individuals to gain access to, understand and use information in ways which promote and maintain good health’ by the World Health Organization (World Health Organization [WHO], 1998, p. 10). According to Nutbeam (2000), health literacy is not simply the act of applying the concept of literacy to a health context, but instead, the comprehensive embracing of ‘critical analysis’, ‘advocacy’, and ‘empowerment’ (Alfrey & Brown, 2013). That is, students with complete health literacy have a capacity to understand, qualify, and utilize health information to promote empowerment of individuals and the community as a whole (Nutbeam, 2000).

In South Korea, academic research on elementary school students’ health literacy is still in its infancy. Ahn and Kwon (2014) measured the linguistic level of elementary school students’ health literacy through questionnaires and reported that their health literacy was very rudimentary. Also, Hong, Shin, and Chang (2017) developed an assessment tool to measure oral health literacy of fifth and sixth-grade elementary school students. This research laid a cornerstone for academic interest in the health literacy of elementary school students in South Korea; however, the studies have limitations in that they focused almost entirely on the development of knowledge and skills for health literacy. Moreover, since most of the research on health literacy in South Korea has been conducted in the medical field (Kim, Oh, & Lee, 2013), there has been little attention paid on program development to nurture children’s health literacy in elementary physical education, specifically.

Although literature has conceptualized health literacy as a multidimensional concept (Alfrey & Brown, 2013), health literacy is currently still regarded as a cognitive and functional term and one that does not embrace physical activity as a core component. Physical activity is an indispensable factor in healthy lifestyles as it provides crucial opportunities for students to improve their health. Students’ active participation in physical activity should be promoted as the key aspect of health literacy. This is especially true in the context of today’s Korea, being that health management capacity is presented as one of the overarching goals stated in the KNCPE.

Therefore, the present concept of health literacy should be supplemented in a way that physical activity, the key sector of physical health, could be regarded as being equivalent to other cognitive and social skills needed for healthy lifestyles. In this research, the term ‘physical activity health literacy’ is defined more broadly and tentatively as a ‘whole capacity to promote and sustain healthy life in functional, cognitive and affective aspect with consistent participation into physical activity’.

The purpose of this research was s to implement a LEAD (Literate, Empowered, Active, Doer) health program to promote the physical activity health literacy of Korean elementary school students by incorporating physical activity, physical fitness and health information, and to examine the impacts of the program. Specific questions are: (a) what are the impacts of the LEAD health program on children’s physical activity health literacy? (b) what are the characteristics of the LEAD health program that influenced the children’s physical activity health literacy enhancement?

## Methods

### Context and setting

A 12-week physical activity health literacy program called ‘Literate, Empowered, Active, Doer’ (LEAD) was designed. The program was developed with the help of students and teachers based on needs analysis. Furthermore, it was also reviewed by PE and health specialists. It includes (a) *PE Lesson*, (b) *Health Tree*, (c) *Health Calendar*, and (d) *Health Journal*. All the components of the program were centred on developing ‘holistic health’, ‘physical activity’, ‘health information’, and ‘a healthy environment’.

To begin with, forty-minute PE lessons were conducted once a week to provide students with information on the concept of health, physical fitness such as cardiovascular endurance, and other main components of healthy lifestyles, such as nutrition, hygiene, and safety. Each PE lesson consists of various activities to develop cognitive, functional, and affective aspects of healthy lifestyles, including physical activities.

Second, the Health Tree was designed to provide open access to health information with students and nurture their critical literacy for authentic information. Each student searched for useful health information via prompts (e.g., ‘the reason why we should eat breakfast’) and shared it on the Health Tree by posting their responses. The Health Tree was set up in the classroom as a poster format, and the students assessed its information, providing critical comments or responses to other students’ inclusions.

Third, the Health Calendar was designed to promote healthy decision-makings and behaviours in students’ everyday lives. Using the Health Calendar, groups of students included specific healthy behaviours to adopt that would serve to strengthen their own physical, social, and mental health. In this process, they were encouraged to utilize various materials including fitness equipment, such as yoga mats and gym balls, which were placed in the classroom. They checked ‘O (good)”, “△ (so so)” and “X (bad)” to show their degrees of achievement each day.

Fourth, the Health Journal was adopted to develop children’s reflective thinking and track their internal changes. The students were asked to write the Health Journal to record newly acquired knowledge, functions, attitudes, and reflections on their own health status. They were asked to write an entry in the journal at least once a week after PE class by the classroom teacher, and they also used it when taking health information notes.

### Ethical considerations

This research was approved by the authors’ University Institutional Review Board (SNU 17–04-013) after the authors had completed the Collaborative Institutional Training Initiative Program prior to the implementation of the research. The research met the ethical principles for medical research on human subjects set out in the declaration of Helsinki (WMA, 2013). The research fulfilled requirement on information, consent, confidentiality and safety of children and guided by ethical principles of autonomy, beneficence, non-maleficence, and justice. Informed verbal and written consents were collected from the administrator of the elementary school, the children, and the legal representatives of the children. The informed consent included information on purpose and procedure of the study, risks and benefits, right to withdraw at any time without any explanation, and confidentiality.

### Participants

The LEAD program was implemented with17 fifth grade children (7 girls, 10 boys; 12 years old) and among them, six fifth grade elementary school students (3 boys, 3 girls) were selected based on their genders, as well as their specific levels of physical activity participation. The backgrounds of the children are presented in Table I.

**Table I.** Background information of the participants

No	Name	Gender	Characteristics
1	Nahyun	F	Very negative about her body image
2	Sarang	F	Self-expressive and active in PE class
3	Yoona	F	Introverted personality with a high level of physical skills
4	Taemin	M	Lacks confidence in his physical skills and exhibits a lower level of physical activity
5	Jisung	M	Optimistic personality and enjoys physical activity
6	Junho	M	Shows active participation in physical activities

### Data collection

Data were collected from (a) open-ended questionnaires, (b) in-depth interviews, (c) student journals, and (d) the observation of PE classes.

Open-ended questionnaires were administered both before and after the program implementation to examine the changes of the children’ experience throughout the program. These included the children’ understanding of health and fitness, the practical efforts necessary for healthy lifestyles, and their overall responses to the program. The in-depth interviews were conducted individually to corroborate the crude data obtained from the open-ended questionnaires. To elicit precise responses from the interviewees, pictures taken and journals written during the program were used with the interviews. Table II shows interview questions that were asked of the children.

**Table II.** Interview questions for the children

Phases	Questions
Pre-Implementation	1. Draw and describe a healthy person. 2. On a scale from 1 to 10, how healthy do you think you are? Explain your response. 3. Have you heard of physical fitness? Tell me five words related to physical fitness and explain them to me. 4. What do you usually do during your break time/lunch time at school? 5. What do you usually do in your spare time at home? 6. Where do you get information on health? 7. Do you use this information on health? If you do, when?
Post-Implementation	1. Tell me about the most impressive experience you encountered during the program. 2. Do you think this program is different from the PE class you attended before? If so, why? 3. Draw and describe a healthy person. If something has changed from your first drawing at the start of the program, what is it? 4. On a scale from 1 to 10, how healthy do you think you are? If your health level is better or worse than you answered before taking the program, why is that? 5. Which part of the program do you think was most helpful to your health? 6. Now that you’ve finished the program, what do you usually do during your break time/lunch time at school? 7. Now that you’ve finished the program, what do you usually do in your spare time at home? 8. Now that you’ve finished the program, how do you get and use information about health?

In the Health Journal, children were asked to express their experience of the program in various aspects including what they learned and felt throughout the program. Finally, the 12 weeks of observation was conducted by the first author during school hours to collect various data about the perceptible changes in lifestyle, including specific behaviours and verbal expressions from the children, both in PE class and in the rest of school life. The observation was recorded in field notes and the informal interview was conducted after the observation; both were subsequently referenced whenever clarification was needed.

### Data analysis

Qualitative content analysis (Graneheim, Lindgren, & Lundman, 2017) was used to analyze data based on inductive approach. First, the transcription of the collected data from the open-ended questionnaire, in-depth interviews, journals from the children, and observation field notes were conducted and reviewed repeatedly by the researchers. While reading the data, the researchers made analytic memos that can be helpful for initial analysis. Then, the researcher segmented important parts of the transcribed data and assigned meaning units. The meaning units were condensed and compared to identify whether there were any similarities or patterns among them. Subthemes were created based on the comparison, and they were reviewed separately by two researchers. If there were any discrepancies, the consistency was checked until they reached a consensus. Finally, the contents of subthemes were integrated and developed into descriptive themes. Table III shows an example of data anlaysis procedure.

**Table III.** An example of data analysis

Meaning unit	Condensed meaning unit	Subtheme	Theme
- “I selected an activity that I could complete with my friends.” - “Time needed for us to try out health information was one of the criteria to select good health information.” - “As I know I have really bad eating habits, I just wanted to try out health information about them.”	- An activity that I could complete with my friends - Time needed for us to try out health information - Health information about bad eating habit	- Establishing criteria	Being a smart finder of health information

### Establishing trustworthiness

In order to ensure trustworthiness of research, various approaches were adopted. First, to ensure credibility of data, the researchers developed an early familiarity of Korean elementary school setting and culture of children (Shenton, 2004). In addition, data source triangulation was conducted to check the consistency among data collected from open-ended questionnaires, in-depth interviews, and field notes from observation (Guba, 1981; Tracy, 2010). Member checks of data and initial interpretation were conducted by sharing transcripts and initial interpretation with children.

In order to ensure transferability, the researchers provided detailed description on the content and approach of the LEAD program and characteristics of children so that the findings of this can be transferred to similar contexts. Also, during the three peer debriefing sessions, a group of specialists majoring in sport pedagogy checked the overall procedure and validity of tentative conclusions of the study (Shenton, 2004; Tracy, 2010).

## Findings

The findings of this study are presented based on two research topics: (1) children’s experiences of the impacts of the LEAD health program on Korean elementary school children and (2) characteristics of the program that influence children’s physical activity health literacy enhancement. All names used are pseudonyms.

## Children’s experiences of the impacts of the LEAD health program

The findings demonstrated that the LEAD program promoted children’s physical activity health literacy in four categories: being a smart finder of health information, being an active agent for sharing and applying health information, valuing and extending physical activity beyond PE class, and understanding health as a holistic concept.

### Being a smart finder of health information

The pre-implementation interview showed that children had limited knowledge on health information. For example, Sarang said, “When I once searched for some information on YouTube, I came across subjects like ‘Tips for Losing Weight’ in the ‘Recommended Videos’ section.” Children could easily find various kinds of information on website, however, to become a smart finder of health information, they learned the necessity to investigate the source, specifically how to discern reliable sources from unreliable ones. This discerning eye for health information was fostered through delicate cognitive process to assess relevance and validity of the information.

To foster this capability, children practised searching for qualified, credible information from the website at the computer lab. Using various measures such as the Internet, school newsletters, and other types of publications, the children identified useful health information from a plethora of miscellaneous and/or unreliable information. As a result, the children obtained basic connoisseurship to discern authentic and useful health information. They also established several pertinent criteria for identifying useful and credible health information. As Nahyun reported in her journal, when searching for health information, the children used keywords related to physical fitness, nutrition, hygiene, and safety based on the program’s content.

When I entered a website to search for health information, I selected an activity that I could complete with my friends. Also, I learned that it takes time to assess health information. This became a criterion to select good health information.

As presented in Nahyun’s journal, whether or not the children could practice health information with sustainable time and effort in their lives was an important criterion in the children choosing the health information.

After they searched health information individually, the children shared the health information with one other on the Health Tree in the classroom. Consequently, this activity provided numerous examples of health information to other children throughout the program. Since they were asked to add health information on the Health Tree using their own criteria, they learned about new health information from others and exchanged some critical comments on health information with their friends. When asked what he has learned from the program, Taemin mentioned:

Through the LEAD program, I gained more knowledge than I imagined I would. For example, I learned about when I should drink water and when I should eat fruit, and how to practice physical activities for a healthy life.

Children came to understand the concept of health information and learned health information on various issues. The ability to obtain and discern health information makes it available for children to be smart finders of health information.

### Being an active agent for sharing and applying health information

Health Tree is not only a bulletin board with health information but also the place where social interaction took place among the children. The children individually retrieved health information from various sources and then shared the information on the Health Tree. The children were highly attracted to sharing their health information with friends via the Health Tree. As a result, children who were not interested in health information initially came to show active participation in the Health Tree. Regarding the utility of shared information from the Health Tree, Junho stated in his journal:

I gained a lot of health information from the Health tree. For example, sometimes I used to be worried about high blood pressure, so I searched for health information on it from the Health Tree. Before the program, I kept worrying, but now I know there is much more information I can use.

Likewise, children shared health information obtained from the Health Tree with family members. For example, Taemin said, “I share health information with my family members from time to time, saying ‘If you do this, you will be much healthier’!”. Moreover, children believed that their active sharing of health information helped them manage their health habits more effectively. Regarding this, Sarang said, “I posted the health information on the refrigerator and tried it out. Usually, I look around the kitchen every day when I come back home.”

### Valuing and extending physical activity beyond PE class

The children learned various physical activities to enhance health-related fitness (e.g., muscular strength, muscular endurance, cardiovascular endurance, flexibility) and exercise-related fitness (e.g., balance, explosive muscular strength, agility, coordination) through PE classes, as a part of the program. Based on what they learned, children made a weekly plan called the Health Calendar, which contains what, where, when, and how to conduct healthy behaviours in school life. Then, they practised their planned physical activities and related healthy behaviours, tracking the number of days they participated in physical activities.

As a result, a number of the children reported that they participated in physical activities more actively than before, not only in PE class but also in their school lives and daily lives, too. For example, Yoona said:

We did physical activity as we planned in our Health Calendar at the gym or outside the classroom during our lunch time. (…) Things have changed. We used to play board games, sitting around, but now we have become more active.

Children had recognized their free time in school just as ‘playtime’ before the program. But, as they actively participated in physical activities scheduled on the Health Calendar throughout the program, they evaluated their school day more productively than before. Furthermore, some children described that they became more open to physical activity. For example, Nahyun reported:

I can enhance my cardiovascular endurance with indoor exercises. I once thought cardiovascular exercises would be really hard, but the truth was beyond my expectations. They can be simple–and even like games. I can do these sorts of exercises with my family members and friends at the playground.

Children realized they could strengthen their physical fitness anywhere, using various physical activities. Some children reported that they tried simple indoor physical activities such as stretching and free weight training with their family members at home.

During the summer vacation, the children made individual Health Calendars as they did in school. They applied what they had learned in the PE class into their everyday lives outside school, doing a number of physical activities consistently. When she was asked about the summer vacation Health Calendar, Sarang mentioned:

Frankly speaking, sometimes it was annoying for me to exercise. (smile) (…) But I worked out every day before I went to sleep. You see, sit-ups, from here to here … (pointing out to one part of the Health Calendar). I even did it at summer camp.

Although the environment was not supportive for her to do physical activities, Sarang overcame the practical barriers and persisted by doing the very physical activities that were planned in the Health Calendar. As Sarang did, the children extended a number of physical activities that they had learned in the program beyond school.

### Understanding of health as a holistic concept

In the first lesson of PE class, the children were asked to draw a healthy person as a group. They shared the idea with their group members and drew a healthy person based on the shared ideas. After they drew a healthy person, they explained the details of their drawings. Picture 1 shows the children’ preconception of what constitutes ‘a healthy person’.

**Figure 1.**
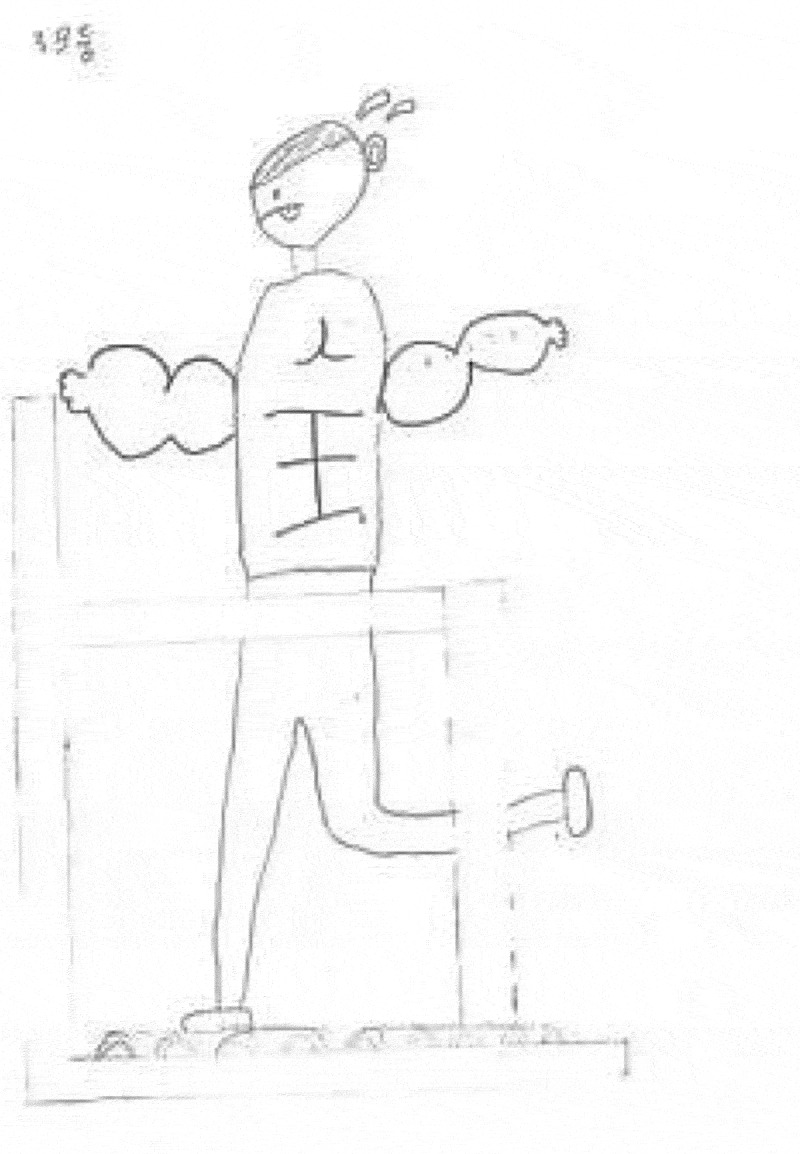
Children’s drawing of a healthy person before the program.

As evidenced in figure 1, the children drew a tall and muscular man running on the treadmill, which indicates that their concept was narrowly circumscribed within physical health. Additionally, when the children were asked whom they would choose for a healthy person, Nahyun said, “I think a healthy person would look very strong, for example, they have lots of muscle and power”.

Since they were unaware of the meaning and importance of multidimensional health, the concepts of physical, social and, mental health needed to be given to them throughout the program. The children were provided with various examples related to physical, social, and mental health (e.g., a girl with a good body shape who suffers from depression), as certain medical conditions could be too abstract and complicated for the children to understand.

Also, regarding the Health Calendar, children were asked to practice healthy behaviours to promote not only physical health, but also social and mental health, such as ‘cheering up friends when working out’ and ‘being positive when dealing with difficulties’. As a result, the children could recognize the meaning and importance of multifaceted health throughout the program. For instance, when she was asked about what makes a healthy person, Sarang said:

Before the program, I thought ‘a healthy person’ meant a person who is just good at sports. But now I think a healthy person should be healthy not only physically but also mentally. I realized that being a healthy person is not easy at all. (…) And I think Jihee is the healthiest person in our class, because she is good at sports, has a lot of friends, always speaks politely to others, and takes care of her friends all the time.

**Figure 2.**
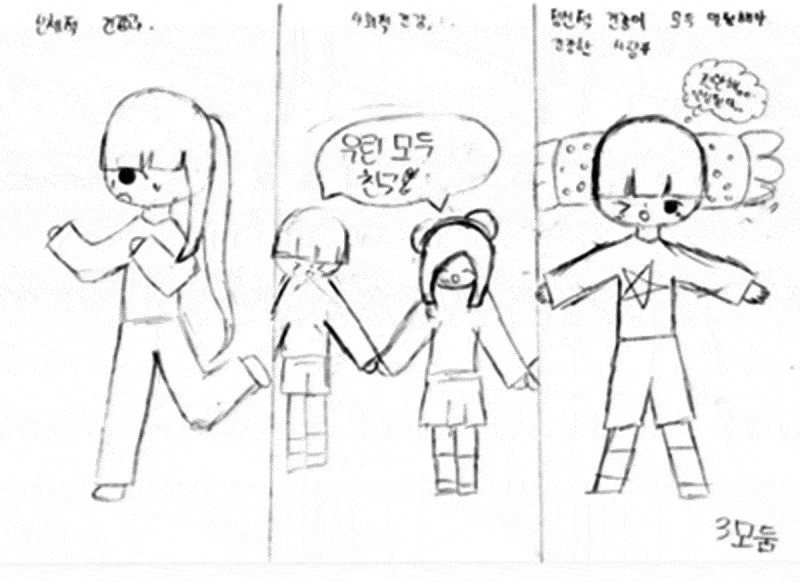
Children’s drawing of a healthy person after the program.

Children’s perception of health shifted from physical health to holistic health upon completion of the LEAD program. Figure 2 demonstrates that the children gained an understanding of healthy concepts in a more comprehensive manner.

As illustrated in Figure 2, the children drew and described three aspects of a healthy person: a girl jogging for physical health, hanging out with others for social health, and relaxing for mental health. The children concluded that physical health alone cannot solely constitute a healthy lifestyle, even though it is an important part of constructing a healthy lifestyle.

## Characteristics of program influencing Korean children’s health literacy

There are three characteristics of the LEAD program that had major influences on the enhancement of elementary school children’ physical activity health literacy. These included: providing specified contents with various physical activities, granting autonomy to children, and building a supportive environment.

### Providing specified contents with various physical activities

Three aspects of the educational contents from the LEAD program affected the elementary school children’ physical activity health literacy development. First, the children’ understanding of health was improved through the introduction of knowledge which was tailored to elementary children’ developmental capabilities. Since the terms related to health, especially physical fitness, were unfamiliar to elementary school children, the meanings were described in easier ways for them to understand. When she was asked about PE class, Yoona reported:

I learned some new words, like cardiovascular endurance, muscular endurance, and muscular strength. (…) When we were learning the new terms, the teacher explained the simple meanings of them, and used many photographs and videos before we started physical activities.

Given that the concept of physical fitness is too abstract and complicated for elementary school children to understand, various visual aids were provided to strengthen children’ learning of health and fitness concepts.

Second, as the knowledge of health and fitness was defined as a variety of physical activities, the children could obtain a more sophisticated understanding into what they were learning. For instance, Junho said:

Now I know the exact meaning of physical fitness, and I think doing a lot of physical activities helped me understand physical fitness. We did a lot of physical activities when we learned about physical fitness in PE class. If we learned about physical fitness without being given the examples of physical activities, it would be much harder for me to understand.

In order to have a clear understanding of the physical fitness concept, children should perform diverse fitness-enhancing physical activities themselves. Additionally, the idea of performing a number of different physical activities attracted children’ attention, thus making them more interested in the LEAD program. Some children indirectly expressed a negative attitude toward other classes only known for delivering surface-level knowledge. For example, Sarang said:

We learned about a lot of different activities in this program, so it wasn’t boring. To be honest, I would fall asleep if I sat all day long in school. Now I can keep moving and running with my friends thanks to this program, and it’s fun and enjoyable.

Third, the children obtained a ‘deep understanding of the concept of physical fitness’. This powerful characteristic is influenced on effective implementation of the program. When she was asked about factors of the program which had influenced the development of her personal health literacy, Yoona answered:

I think we all have to know physical fitness in detail. If we know physical fitness in detail, it’s easy for us to exercise, and also we will be eager to do it. If we don’t know it in detail, we cannot exercise even if we want to.

The scope and depth of knowledge provided with children had a great importance in transferring physical activity outside of physical education classes. In fact, the knowledge of various physical activities can give a positive impetus for children who are not motivated internally.

### Granting autonomy of learning to children

There are also several features of methods used in the LEAD program that influence the physical activity health literacy of elementary school children. First, the student-centred cooperative learning provided consistent opportunities for children to experience various kinds of problem-solving, and they experienced a sense of joy derived from the collaboration. For example, Jisung said:

I think, when we are doing exercises, sharing our feelings with friends is important. When we share our feelings and thoughts with others, we can get much closer. We can cooperate with unfamiliar friends when exercising together.

As Jisung mentioned, developing peer relationships through group activities consistently promoted social interactions between the children, a factor that became key to their active participation as a group. This is especially true in the sense that children had to share ideas with their friends in order to plan the Health Calendar. By doing this, they experienced the importance of social interaction in helping make every group member physically motivated.

Also, they valued their empowerment obtained through the LEAD program since they could create and organize various activities by themselves in school life. Junho reported:

We used to do whatever the teacher asked us to do both during class time and break time with no plan. But, now, we can plan what we will do as we want, using what we learned in class. I think that’s the best change from before the program.

Although children knew it was unproductive, they spent their break time just for chatting with friends before implementing the program. However, given the new autonomy in their own school lives, they could choose what, where, when, and how to conduct their lunchtimes and break times based on what they learned in PE class. This experience made children more interested in self-determining a healthy school life.

Moreover, given the fact that children had opportunities to reflect on their own health status and practice during this program, as opposed to the numerical outcome assessed by the teacher, children made quite objective and exhaustive evaluations on themselves, an occurrence which ignited the motivation for a healthier life. Junho said:

I think writing a health journal is the most useful activity for me in this program as it can allow me to express my feelings and opinions freely from PE class and the Health Calendar. I can see what I recently learned and think through it while writing a journal entry.

As Junho mentioned, looking back on children’ own health statuses and identifying what they learned and missed through the program strengthened their will for healthy behaviours.

### Building a supportive environment

The environmental features affecting the elementary school children’ physical activity health literacy improvements could be subdivided into physical environmental features and psychological environmental features. The physical environmental features include sporting equipment and the Health Tree, along with various health information labelled throughout the classroom. Regarding the clear differences between average PE classes and the LEAD health program, Sarang said:

Sometimes writing health information down is annoying, but I know I can find some good health information (from the Health Tree) and try it out. And the Health Calendar also has a few merits. I get refreshed after doing exercise though it is hard for us to get it done in the way we planned in the Health Calendar … We have some yoga mats and a gym ball here in the classroom, so we can work out whenever we want to.

The sporting equipment available for classroom physical activity was a supporting factor for their increased physical activity participation. Also, the Health Tree in the classroom was another main factor that influenced the children’ improved acquisition and utilization of health information.

As for the psychological environmental features, the permissive atmosphere encouraged the children to overcome difficulties involved in the program and stick with their healthy behaviours. This consideration is a far cry from traditional techniques in which children are compared and ranked for competence. In terms of the psychological atmosphere, Sarang reported:

When we were stretching our legs, Hyeyoon and Sarang pushed my back down slowly and softly, because stretching really hurts for me. Before the program I once had thought exercising was just kind of exhausting hard work, but now it has become fun and interesting, thanks to my friends

Children with inactive proclivity and unsophisticated sporting skills tried to get over the physical and emotional barriers when given consistent support and encouragement from friends.

## Discussion

This study investigated Korean elementary school children’ physical activity health literacy developments while participating in the LEAD health program and identified specific characteristics of the program influencing their health literacy development. As for findings, four aspects of elementary school children’ physical activity health literacy were developed through the LEAD health program: being a smarter finder of health information, being an agent for sharing and applying health information, valuing and extending physical activity beyond PE class, and understanding health as a holistic concept.

Above all, the findings demonstrated that the children’s improved health behaviours were attributed to environmental factors. This indicates a strengthening in each individual’s personal endeavour, but also the fact that a supportive sociocultural environment should be provided to enhance the health literacy of elementary school children. In previous research, the importance of supportive environments has been emphasized to promote children’s healthy lifestyles. According to Albert and Steinberg (2011), adolescents’ decisions to participate in health behaviours are considerably susceptible to socio-emotional factors (Fleary, Joseph, & Pappagianopoulos, 2018), particularly the peer group. The peer group, in particular, has been argued as one of major factors affecting adolescents’ health literacy and health behaviour development (Manganello, 2007). Additionally, Lee and Jeon (2012) also suggested that health-oriented school environments are essential for effective implementation of health promotion programs. Thus, in order to enhance elementary school student’s physical activity health literacy and healthy behaviours, there should be concerted efforts made by the children themselves, health policy providers, as well as special attention paid to the school settings (Manganello, 2007).

Additionally, in this research, children obtained a discerning ability to analyze health information critically and utilized it in their real lives throughout the LEAD health program. They established their own criteria to distinguish useful health information from unreliable health information and shared the selected information with their friends and family members. In previous studies Nutbeam (2000, 2008) presented critical health literacy as one of the core concepts of health literacy, and Fleary et al. (2018) noted that learning how to extract essential health messages from raw health information is vital for adolescents in developing critical health literacy and healthy behaviours. Given that a majority of research about health literacy is concentrated on functional health literacy and critical health literacy, it has rarely been investigated in the school context (Fleary et al., 2018). That said, this research serves to provide practical information needed for elementary school children’ critical health literacy development. Moreover, given that a number of adolescents have used various kinds of mass media, including Internet websites as instruments to obtain health information (Ghaddar, Valerio, Garcia, & Hansen, 2012; Manganello, 2007), the LEAD children have now learned how to identify reliable health information on the Internet. This fact stands as robust evidence for early adolescents’ media literacy development.

Finally, this research proves a strong possibility that programs for physical activity health literacy development can be implemented effectively as a main part of the ‘health’ area in KNCPE. It is often reported that there is a considerable discrepancy between what is originally intended in health curriculum and what is actually implemented in school (Alfrey & Brown, 2013), primarily because the health domain in KNCPE seems to be misinterpreted in its practice. Although the health domain is one of the key portions of the KNCPE, the hygiene, nutrition, and safety sections of the health domain are frequently regarded as monotonous and uninteresting because they remain separated from physical activities (Park, 2017). Health-literate individuals can read meaningful health messages from information and make their own lives more health-oriented with well-informed decision-making (McCuaig, Carroll, & Macdonald, 2014; Shohet & Renaud, 2006). By giving children the access they need to make informed decisions, we can, ultimately, ameliorate current health risks common in Korean society today.

Although physical activity health literacy is a relatively unfamiliar concept to PE educators in South Korea, literature notes that health literacy has become one of the core elements in the national curriculum of health and physical education worldwide (Australian Curriculum, Assessment and Reporting Authority, 2013; McCuaig et al., 2014). Therefore, it is worthwhile to nurture health-literate children and fairly deliberate physical activity health literacy as one of the national standards of physical education in South Korea. Thus, various health literacy enhancement programs should be developed to be implemented in diverse contexts, specifically in each level of school. Through this initial research, based on ‘health management capacity’ presented in ‘KNCPE’, the LEAD health program was designed and conducted to develop the holistic aspects of physical activity health literacy, including active participation in physical activity throughout all facets of school life. The findings of this study will serve as a practical platform in developing health curriculum for elementary school children in the future.

## Limitations of the study

This study has limitation because only six children were selected as participants which limit the scope of interpretation and transferability (Graneheim & Lundman, 2004). Given that the 12-week LEAD health program used in this research was not a long-term project, there is a possibility that more diverse and unexpected changes can be detected as lasting effects from the children. Furthermore, as changes in children’s everyday lives are measured by relying on their own reports in this research, longer implementation of the program would make it possible for researchers to obtain more reliable evidence.

To overcome these limitations, physical activity health literacy studies in the future should be conducted as a longitudinal study, collecting data by both qualitative and quantitative methods. As for physical fitness, especially, an increase in quantitative measurement will serve as robust evidence for children’s improvement in physical fitness. Additionally, data concerning children’s sustaining healthy lifestyles should be collected consistently by questionnaires to examine the lasting effects of the LEAD health program.

## Conclusion

The purpose of this study was to describe children’s experiences of the impact of LEAD health program on their physical activity health literacy and to describe the characteristics of the LEAD health program that influenced the children’s health literacy enhancement.

As a result, children learned how to select and obtain health information, expanded their notion of health from physical health to holistic health, extended their participation in physical activities from PE class into their everyday lives, and, most importantly, experienced the value of physical activity. The conceptual and behavioural changes of the children indicate that the LEAD health program can be an effective way for children to develop the knowledge, skills, and attitude necessary to maintain a healthy lifestyle into adulthood. Additionally, the program helped children to obtain a strengthened and informed health management capacity, which, after all, is main goal of the health area in KNCPE. In addition, the influential program characteristics identified in this study such as providing specified content on various physical activities, granting autonomy to children, and building supportive environments can be practical guidelines for designing future programs.

## References

[CIT0001] Ahn E. J., & Kwon I. S. (2014). Health literacy of elementary school students. *Child Health Nursing Research*, 20(4), 322–11.

[CIT0002] Albert D., & Steinberg L. (2011). Judgment and decision making in adolescence. *Journal of Research on Adolescence*, 21(1), 211–224.

[CIT0003] Alfrey L., & Brown T. D. (2013). Health literacy and the Australian curriculum for health and physical education: A marriage of convenience or a process of empowerment? *Asia-Pacific Journal of Health, Sport and Physical Education*, 4(2), 159–173.

[CIT0004] Australian Curriculum, Assessment and Reporting Authority (2013). *Australian curriculum: Health and physical education*. Sydney: Author.

[CIT0005] Ereaut G., & Whiting R. (2008). What do we mean by ‘wellbeing’? And why might it matter? (Department for Children, Schools and Families, Research Report No DCSF Research Report DCSF-RW073). UK.

[CIT0006] Fleary S. A., Joseph P., & Pappagianopoulos J. E. (2018). Adolescent health literacy and health behaviors: A systematic review. *Journal of Adolescence*, 62, 116–127. 29179126 10.1016/j.adolescence.2017.11.010

[CIT0007] Ghaddar S. F., Valerio M. A., Garcia C. M., & Hansen L. (2012). Adolescent health literacy: The importance of credible sources for online health information. *Journal of School Health*, 82(1), 28–36. 22142172 10.1111/j.1746-1561.2011.00664.x

[CIT0008] Graneheim U. H., Lindgren B. M., & Lundman B. (2017). Methodological challenges in qualitative content analysis: A discussion paper. *Nurse Education Today*, 56, 29–34. 28651100 10.1016/j.nedt.2017.06.002

[CIT0009] Graneheim U. H., & Lundman B. (2004). Qualitative content analysis in nursing research: Concepts, procedures and measures to achieve trustworthiness. *Nurse Education Today*, 24, 105–112. 14769454 10.1016/j.nedt.2003.10.001

[CIT0010] Griggs G. (2015). *Understanding primary physical education*. London: Routledge.

[CIT0011] Guba E. G. (1981). Criteria for assessing the trustworthiness of naturalistic inquiries. *Educational Communication and Technology Journal*, 29, 75–91.

[CIT0012] Hong J., Shin Y., & Chang K. (2017). Development of oral health literacy assessment tool for fifth and sixth grade elementary school students. *Journal of Korean Academy of Oral Health*, 41(3), 214–221.

[CIT0013] Kickbusch I., Pelikan J. M., Apfel F., & Tsouros A. (2013). *Health literacy: The solid facts*. Copenhagen, Denmark: World Health Organization Regional Office for Europe Retrieved from http://www.who.int/iris/handle/10665/128703

[CIT0014] Kim S., Oh J., & Lee Y. (2013). Health literacy: An evolutionary concept analysis. *The Journal of Korean Academic Society of Nursing Education*, 19(4), 558–570.

[CIT0015] Korean Ministry of Education (2015). *2015 national curriculum for physical education*. Seoul: Ministry of Education.

[CIT0016] Korean Ministry of Education (2018). *Statistics on student health test in 2017*. Retrieved from https://www.gov.kr/portal/ntnadmNews/1387391

[CIT0017] Lee K. (2010). The policy of health education in the physical education of 2007 revised elementary school curriculum. *The Korean Journal of the Elementary Physical Education*, 16(2), 1–8.

[CIT0018] Lee O., & Jeon S. (2012). The practice and developing in-school PE programs for elementary school girls’ health and fitness. *The Korean Journal of Elementary Physical Education*, 17(4), 143–156.

[CIT0019] Lee Y. (2012). A study on relevance between health-related physical fitness and obesity in ‘working-out elementary students to boost physical activities. *The Korean Journal of the Elementary Physical Education*, 18(2), 223–234.

[CIT0020] Manganello J. A. (2007). Health literacy and adolescents: A framework and agenda for future research. *Health Education Research*, 23(5), 840–847. 18024979 10.1093/her/cym069

[CIT0021] McCuaig L., Carroll K., & Macdonald D. (2014). Enacting critical health literacy in the Australian secondary school curriculum: The possibilities posed by e-health. *Asia-Pacific Journal of Health, Sport and Physical Education*, 5(3), 217–231.

[CIT0022] Nutbeam D. (2000). Health literacy as a public health goal: A challenge for contemporary health education and communication strategies into the 21st century. *Health Promotion International*, 15(3), 259–267.

[CIT0023] Nutbeam D. (2008). The evolving concept of health literacy. *Social Science and Medicine*, 67(12), 2072–2078. 18952344 10.1016/j.socscimed.2008.09.050

[CIT0024] Park M. (2017). *How healthy is the health education in elementary school?: A pedagogical exploration for the effective teaching of health activity in elementary school*. (Unpublished doctor’s thesis) Seoul National University, Seoul, South Korea.

[CIT0025] Rhee M., & Jo K. (2015). The analysis of research trends related to health and fitness and exploring future directions in primary physical education. *The Korean Journal of the Elementary Physical Education*, 21(1), 211–222.

[CIT0026] Shenton A. K. (2004). Strategies for ensuring trustworthiness in qualitative research projects. *Education for Information*, 22, 63–75.

[CIT0027] Shohet L., & Renaud L. (2006). Critical analysis on best practices in health literacy. *Canadian Journal of Public Health*, 97, S10–S13. 10.1007/BF03405366 PMC6975758 16805154

[CIT0028] Tracy S. (2010). Qualitative quality: Eight “Big-Tent” criteria for excellent qualitative research. *Qualitative Inquiry*, 16(10), 837–851.

[CIT0029] WMA (2013). *World medical association declaration of Helsinki. Ethical principles for medical research involving human subjects*. Ferney-Voltaire, France: Author. 10.1001/jama.2013.281053 24141714

[CIT0030] World Health Organization (1948). *World health organization constitution: Basic documents*. Geneva, Switzerland: Author.

[CIT0031] World Health Organization (WHO) (1998). *Health promotion glossary*. Geneva: World Health Organization.

[CIT0032] Yu X., Yang T., Wang S., & Zhang X. (2012). Study on student health literacy gained through health education in elementary and middle schools in China. *Health Education Journal*, 71(4), 452–460.

